# Improving completion rates of students in biomedical PhD programs: an interventional study

**DOI:** 10.1186/s12909-017-0985-1

**Published:** 2017-08-25

**Authors:** Marin Viđak, Ružica Tokalić, Matko Marušić, Livia Puljak, Damir Sapunar

**Affiliations:** 1 Medical School, University of Split School of Medicine, Split, Croatia; 2 Department of Research in Biomedicine and Health, University of Split School of Medicine, Split, Croatia; 3 Laboratory for Pain Research, University of Split School of Medicine, Split, Croatia

## Abstract

**Background:**

Analysis of graduation success at the University of Split School of Medicine PhD programs conducted in 2011 revealed that only 11% of students who enrolled and completed their graduate coursework between 1999 and 2011 earned a doctoral degree. In this prospective cohort study we evaluated and compared three PhD programs within the same medical school, where the newest program, called Translational Research in Biomedicine (TRIBE), established in the academic year 2010/11, aimed to increase the graduation rate through an innovative approach.

**Methods:**

The intervention in the new program was related to three domains: redefined recruitment strategy, strict study regulations, and changes to the curriculum. We compared performance of PhD students between the new and existing programs and analyzed their current status, time to obtain a degree (from enrolment to doctorate), age at doctorate, number of publications on which the thesis was based and the impact factor of journals in which these were published.

**Results:**

These improvement strategies were associated with higher thesis completion rate and reduced time to degree for students enrolled in the TRIBE program. There was no change in the impact factor or number of publications that were the basis for the doctoral theses.

**Conclusion:**

Our study describes good practices which proved useful in the design or reform of the PhD training program.

## Background

The traditional measures for assessing the success of doctoral education programs have been time to PhD and completion rates [1–3]. However, such information is rarely published, either at institutional, discipline, or country level, making it hard to obtain data on the subject, especially for European PhD programs. The most comprehensive data on completion of PhD programs in the USA and Canada are available from The Council of Graduate Schools (CGS) PhD Completion Project. According to the Project report, which was published in 2008, only 57% of PhD students complete their program within a decade [4].

According to the report on PhD completion rates at Canadian universities, most graduate schools do not even record such statistics and tend to perceive that their PhD programs have higher completion rates and shorter times-to-degree than other universities [5]. Anecdotal information and personal experience leads us to conclude that the completion rates of graduate programs are generally low.

An internal analysis of graduation success in the PhD programs at the University of Split School of Medicine, Croatia, conducted in 2011, revealed that only 11% of students who enrolled and completed their graduate coursework between 1999 and 2011 obtained a doctoral degree. We therefore set up a prospective cohort study to examine the performance of three current PhD programs within the same medical school; among them, the newest program, called Translational Research in Biomedicine (TRIBE), established in the academic year 2010/11, aimed to increase graduation rates through an innovative approach. The aim of this study was to assess the value of strategies implemented in the new PhD program and to compare its success with that of two existing programs within the same institution.

## Methods

### Settings

The higher education system in Croatia incorporates three cycles (undergraduate, graduate and postgraduate studies), in line with the Bologna Process guidelines. The first cycle is a 3-year undergraduate or bachelor degree and the second cycle is a 1 or 2-year master’s degree. In certain professions such as medicine, dentistry, law, veterinary medicine and education, there are 5-year or 6-year integrated undergraduate and graduate university courses. The third cycle includes postgraduate studies, which can be 3-year doctoral (PhD) studies or a specialist studies that lead to professional qualification. Doctoral programs may be started after the completion of either graduate studies or an integrated undergraduate and graduate university course [6].

PhD programs in Croatia are aligned with current European directives [7–9]. They have a standard length of three years and are allotted a total of 180 European Credit Transfer System (ECTS) points.

Currently there are 10 PhD programs in biomedicine in Croatia (5 in Zagreb, 3 in Split, one in Rijeka and one in Osijek) and most of them award doctorates in basic and clinical medical sciences. Dental medicine, pharmacy and veterinary medicine have their own PhD programs. Almost 60% of PhD students are women between 20 and 29 years of age and majority of them are from Croatia. Most of the them study part-time, while employed in the health care system. A smaller number of students are full-time researchers employed by medical schools as a faculty, doctoral students, or as junior scientists working on research grants [10].

According to current University of Split School of Medicine (USSM) bylaws, after three years of PhD program students have an undefined period in which to defend their thesis. In addition to doing independent research, PhD candidates must attend formal courses, pass exams and, most important, be listed as first author on two academic articles (or one if the impact factor of the journal in which they publish is higher than 4). The thesis must be based on original results published in journals indexed in Web of Science (WoS) or Current Contents (CC) databases and have the impact factor ≥ 1. The PhD program is completed by the defense of the thesis. Depending on the PhD program, the USSM charges tuition fees of between €2000 and €3000 per academic year. However, PhD students who are employed at USSM are not required to pay for their tuition and most of the students employed via research grants from the Croatian Science Foundation receive a scholarship for the PhD program from those grants.

**Fig. 1:**
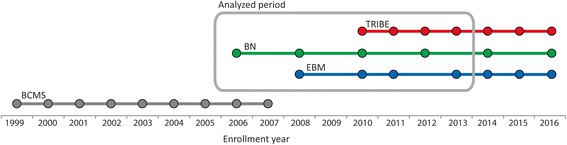
Enrollment history of the PhD programs at University of Split School of Medicine (USSM). Legend: BCMS - Basic and Clinical Medical Sciences; BN - the Biology of Neoplasms; EBM - Evidence-Based Medicine; TRIBE - Translational Research in Biomedicine; Circles represent enrollment events for the particular PhD program

The USSM is located in Split, the second largest city in Croatia (metropolitan area population ≈350,000). It is one of four medical schools in Croatia. The School was founded in 1997, but the medical program started in 1979, when the school was a subsidiary of the University of Zagreb School of Medicine [11, 12]. The doctoral program in Basic and Clinical Medical Sciences started in 1999/2000. It had three separate courses: Clinical Physiology, Sports Medicine, and Clinical Medicine. Since 2008/09, the Clinical Physiology and Sports Medicine courses have stopped enrolling students while the Clinical Medicine course has been transformed into a new program called Evidence-Based Medicine (EBM). The second current program is the Biology of Neoplasms (BN), established in 2006/07, and the third is Translational Research in Biomedicine (TRIBE), started in 2010/11 (Fig. 1) (Translational research is a subset of biomedical research that aims to apply basic science to concrete clinical problems thus translating laboratory science into healthcare improvements). USSM currently enrolls 35–55 postgraduate students each year (BN 20 biannually, EBM 20 and TRIBE 15 per year).

### Intervention

The TRIBE program was established with the aim of increasing PhD program completion rates by implementing improvements related to three domains a) recruitment strategy, b) program regulations, and c) program curriculum (Table 1). Changes to the recruitment strategy included requiring a feasible research plan as a selection criterion and openness to candidates with backgrounds from research fields other than biomedical. The admissions procedure includes an interview in which students present their planned PhD thesis project, as well as an interview with a proposed mentor. At this time point, the plans are not required to be detailed; the criteria for the positive assessment are the overall scientific validity and feasibility of the research under given local working conditions.

**Table 1:** Interventions aimed at increasing the completion rate and reducing the time to obtaining a degree for students in the TRIBE program

Intervention area	Concrete measure	Description of the intervention	Expected effect
Recruitment strategy	Selection criteria	The main enrollment criterion is presentation of a feasible research plan, with realistic funding options, available equipment and proposed mentor.	Students have a defined research topic and a mentor at the time of enrollment
	Interdisciplinary approach to student recruitment	Admission of students with backgrounds from any scientific field, if their targeted research topic is within biomedicine.	Building interdisciplinary teams collaborating on biomedical research
Program regulations	Student progress reports	Mandatory biannual reports as the main indicator of student progress.	Ongoing insight into student progress
	Strict regulations for enrollment into the next year	For each exam (including biannual reports) there are 4 opportunities per year. If students fail or do not attend these exams they have to repeat the year. Each year can be repeated only once. If they fail to pass an exam in 8 terms or 2 years, they are expelled from the program.	Students pass exams within current academic year
Curriculum	Formal education in relevant skills	Introduction of courses in: Lab management, Research skills, Entrepreneurship and transfer of technology, Ethics in research, Communication and presentation skills.	Acquisition of the necessary skills for a successful research career
	Focus on development of research plan	Obligatory completion of a detailed research plan by the end of the first study year, tested by oral and written examinations.	Research plan completely defined by the end of the first year

The existence of the potential research plan allows students to recognize elements of the curriculum that are important for work on their thesis and immediate engagement in teaching and research activities. This has led to a highly selective enrollment process into the TRIBE program; so far, 6 generations of PhD students have been enrolled into the program and each year the number of enrolled students has been lower than the number of available places.

The program includes obligatory biannual student progress reports that are marked against a) scientific quality of the research plan, b) progress in the research, and c) quality of the presentation. For these reports and for any other exam we strictly adhere to the principles under which students can have only 8 attempts to pass during two years of the program; if they fail, they are excluded from the program. Furthermore, in the TRIBE program, students cannot enroll into the higher year unless they have passed all exams or had an approved report in the previous year of the program. The other two programs do not enforce these regulations and students can have an indefinite number of attempts to pass an exam.

Interventions in the curriculum include training in relevant skills (see Table 1 for details) and obligatory completion of the research plan as a requirement for passing the first year of the program (Table 1).

These aspects make a clear distinction between TRIBE and the two other programs. Those programs base their selection process solely on grade point average achieved during medical school and candidates are mainly restricted to those who have studied medicine. Progress reports are not required. The teaching is directed towards acquiring further specialized knowledge in the discipline and not towards general research methodology. Transferable skills teaching (Table 1) is not provided.

### Archival data

Data were obtained from the USSM’s Office for Postgraduate Studies with the permission of the Doctoral School Committee. The Ethics Committee of the University of Split School of Medicine had waived ethics approval for this study. Consent from students for conducting this prospective study was not obtained because consent was waved as part of ethical exemption. Data were extracted for all students enrolled in the three USSM PhD programs before academic year 2014/15 because it was not expected that the students who enrolled after that date could defend their PhD thesis by the time the data were analyzed (Fig. 1). Students were followed-up until March 2016. We also obtained demographic data of enrolled students (age at enrollment, gender, profession, employment, and tuition arrangements).

### Outcome measures

The primary outcome measure for the three cohorts was the proportion of students defending their doctoral thesis. Other success measures were: time to degree (time from enrollment to defense of the PhD thesis, in months), number of published manuscripts on which the thesis is based, and the impact factor of journals in which these manuscripts were published.

### Statistical analysis

Data are presented as mean and standard deviation or median and range. We used the chi-squared test for nominal variables. For continuous variables, nongaussian distribution was tested with the D’Agostino-Pearson normality test followed by a Kruskal-Wallis test or ANOVA. A *P* value <0.05 was considered statistically significant. All calculations were done using Prism software (GraphPad Software Inc., La Jolla, CA, USA).

## Results

### Previous postgraduate programs (1999–2007)

From 1999/2000 to 2007/08 the Basic and Clinical Medical Sciences program enrolled 336 students. According to the current school bylaws, this program is now closed, i.e. students enrolled in that program may no longer apply for thesis defense so data on that cohort can be considered final (Fig. 1). Of the 336 students, 170 were directly enrolled in the PhD program and 166 in the Master’s program. The Master’s program was a prerequisite for the PhD program for students with lower grade point averages achieved during their undergraduate studies. Students enrolled in the Master’s program were those with grade point averages under 4.0 (from a maximum of 5.0), because at that time that was the threshold for joining the PhD program. Of all 336 students, 36 successfully defended PhD theses, representing 10.7% of enrolled students, leaving 298 students who did not obtain a PhD. Among those who successfully defended a PhD thesis, only 9 have been obtained by students who first concluded the Master’s program while 32 students obtained a Master’s degree but did not succeed in obtaining a PhD.

### Students enrolled in the three current PhD programs

Between the academic years 2006/07 and 2013/14, 237 students enrolled in the three PhD programs at USSM, of whom 60% were women and 40% men (Table 2). The median age of enrolled students was 28 years. There were 209 (88%) medical doctors or doctors of dental medicine (MD/DDM) and 28 (12%) students from other professions. Students’ grade point averages from previous university education were significantly lower in the BN program than in the EBM program (Kruskal-Wallis ANOVA with Dunn’s test, *P < 0.001*).

**Table 2:** Characteristics of students enrolled in three PhD programs at the University of Split School of Medicine between 2006/7 and 2013/14

Variable	BN	EBM	TRIBE	Total
No. of enrolled students	81	101	55	237
Male	36 (45%)	39 (39%)	20 (36%)	95 (40%)
Female	45 (55%)	62 (61%)	35 (64%)	147 (60%)
Age at enrollment (median, range)	33 (23–59)	28 (23–58)	28 (24–53)	28 (23–59)
Grade point average (median, range)	3.86 (2.85–4.95)*	4.14 (2.94–4.95)	4.0 (3.0–5.0)	4.0 (2.85–5.0)
Students paying / not paying tuition	40 / 41	40 / 61	23 / 32	167/106
MD or DDM / other profession	76/5	98/3	35/20	209/28
Employed in academia	12 (15%)	15 (15%)	24 (44%)^#^	51 (22%)
Employed in PHC	13 (16%)	14 (14%)	6 (11%)	33 (14%)
Employed in hospitals	51 (63%)	63 (62%)	17 (31%)	131 (55%)
Employed in other sectors	3 (4%)	3 (3%)	4 (7%)	10 (4%)
Unemployed	1 (1%)	3 (3%)	/	4 (2%)
Missing data on employment	1 (1%)	3 (3%)	4 (7%)	8 (3%)
Attrition rate	2 (2.5%)	1 (0.8%)	7 (12.7%)**	10 (4.2%)

*BN* Biology of Neoplasms, *EBM* Evidence Based Medicine, *TRIBE* Translational Research in Biomedicine, *DDM* Doctor of Dental Medicine, *PHC* Primary Health Care

*Significant difference between grade point average of BN and EBM students (Kruskal-Wallis ANOVA with Dunn’s test, P < 0.001)

** Significant difference in attrition rate between three programs; χ ^*2*^ *(2, N = 237) = 13.7, P < 0.05*

^#^ *Significant difference in employment structure of enrolled students; χ* ^*2*^ *(8, N = 237) = 28.9, P < 0.05*

At the time of enrollment, USSM’s PhD students were employed in hospitals (55%, *n* = 131), academia (22%, *n* = 51), primary health care institutions (14%, *n* = 33) or other sectors (4%, *n* = 10). Only 4 students (2%) were unemployed at the time of enrollment. The proportion of students employed in academia was significantly higher in the TRIBE program than in the other two (44% compared to 15% in EBM and BN program; *χ2 (8, N = 237) = 28.9, P < 0.05*).

However, most of the students employed in hospitals also had some form of academic appointment. In addition, during the study period we observed increasing numbers of medical doctors employed in academia leaving primary appointments in basic science departments to start clinical residencies in hospitals. Except for the difference in student employment structure and in grade point averages we did not find any significant differences in the characteristics of students enrolled in the three PhD programs, suggesting that students represented a single pool of applicants. The only important difference between cohorts was the attrition rate. The TRIBE program had a significantly higher attrition rate than the BN or EBM programs (*χ2 (2, N = 237) = 13.7, P < 0.05*).

### Time to degree

The cumulative degree completion rate for the three PhD programs at USSM was 16.7% after 9 years (analyzed until March 2016) (Table 3). After five years of studying, 29.2% of TRIBE students obtained their degree. In the two other programs the percentage of obtained degrees was below 10%, similar to graduation rates of previous postgraduate programs (between academic year 1999/2000 to 2007/2008) (Table 3).

**Table 3:** Cumulative completion rates for students starting University of Split School of Medicine’s PhD programs

PhD program	Cumulative percentage by year of program						
	3rd yr.	4th yr.	5th yr.	6th yr.	7th yr.	8th yr.	9th yr.
BN	1.3 (1.3)	2.6 (2.5)	9 (8.8)	11.5 (11.3)	14.1 (13.8)	16.6 (16.3)	17.9 (17.5)
EBM		3 (3)	5 (5)	7.9 (7.9)		8.9 (8.9)	9.9 (9.9)
TRIBE	20.1 (18.2)	27.1 (23.6)	29.2 (25.5)				
Total	4.8 (4.7)	7.9 (7.6)	11.5 (11)	13.7 (13.1)	14.5 (14)	15.9 (15.3)	16.7 (16.1)

*BN* Biology of Neoplasms, *EBM* Evidence Based Medicine*, TRIBE* Translational Research in Biomedicine

### Students’ scientific output

During the analyzed period (between academic year 2006/2007 to 2013/1014), the 237 USSM PhD students published 61 original scientific articles which were the basis for 38 PhD theses (Table 4). The average number of publications per thesis was 1.7 ± 0.7. In order to test differences between the three cohorts we compared the number of publications on which the thesis was based and the impact factor of the journals in which these were published. In the observed period, TRIBE students published 23 articles and, based on them, achieved 14 doctorates. The number of theses per enrolled student was significantly higher in the TRIBE cohort than in the other programs (*χ* ^*2*^ *(2, N = 237) = 6.5, P < 0.05*). In the same period, students in the EBM program had 18 publications and achieved 10 doctorates while BN students had 20 publications and achieved 14 doctorates (Table 4). There were no significant differences in the number of publications per thesis or the journal impact factor of students’ publications between the three programs. The mean impact factor of journals in which publications stemming from the theses were published was 4 ± 5.7.

**Table 4:** Scientific output of students enrolled in three USSM PhD programs

	BN	EBM	TRIBE	Total
No. of enrolled student	81	101	55	237
No. of defended theses	14	10	14*	38
No. of publications	20	18	23	61
No. of basic science doctorates	7	5	9	21
No. of clinical science doctorates	6	3	4	13
No. of public health doctorates	1	2	1	4
No. of tuition paying /not paying students with degree	6/8	3/7	5/9	14/24

*BN* Biology of Neoplasms, *EBM* Evidence Based Medicine, *TRIBE* Translational Research in Biomedicine

*Significant difference in number of thesis per enrolled student; χ ^*2*^ *(2, N = 237) = 6.5, P < 0.05*

## Discussion

Our findings suggest that six simple interventions in three domains of PhD program organization resulted in significant increases in completion rates and reduced time to degree. Although PhD degrees earned faster do not necessarily indicate better education, these outcomes are important given the concerns about the duration of PhD studies and research leading to the successful completion of a PhD program in many countries [13].

Delays in obtaining a doctorate are perplexing since students enrolling in PhD programs are a highly motivated population [14, 15]. Our study of three PhD programs found that the students had similar characteristics at enrollment so the observed results in the TRIBE cohort were not a consequence of enrolling different types of student but other factors, contained in the reform.

Students with a strong institutional support structure are more likely to be able to complete their programs. Performance may be affected by support at departmental level, by mentors or role models at the school [16]. However, the three PhD programs analyzed here belong to the same School, with the same institutional support and personnel, so we cannot expect that institutional support is the reason for the different results achieved in the TRIBE cohort.

One limitation of our study is that we do not know which of the six interventions contributed most to the observed results. Based on a previously published study in which we showed that stricter regulations were associated with better academic outcomes for medical students, [17] we assume that the most important interventions were related to more stringent study regulations in the form of mandatory progress reports, obligatory completion of a detailed research plan by the end of the first study year, higher year enrollment regulations and the possibility of being expelled if exams were not passed within two years.

Entrance examinations such as the Graduate Record Examination (GRE) are not required for the three PhD programs at USSM, since there is growing evidence of poor correlation between GRE and ultimate student success [18, 19]. The admissions procedure for the TRIBE program includes an interview in which students have to present the feasibility of their proposed research project and an interview with a potential mentor. Developing a potential research plan allows students to recognize elements of the curriculum that are important for realization of their thesis and immediate engagement in teaching and research activities. The important element for admission in the other two programs was grade point average yet, in spite of this, the TRIBE students did not differ in point grade average from students enrolled in the other programs, which additionally suggests that this factor did not account for the different success rate.

The few studies that have systematically evaluated the impact of various curricula have failed to show any substantial difference between them at least at the undergraduate level [20]. Therefore, we believe that implementation of courses related to complementary skills were probably less important for observed outcomes, although more research would be required to evaluate their contribution to the program success.

Unlike the two other programs, the TRIBE program accepts many students from backgrounds other that medicine. This interdisciplinary approach was based on a principle of openness to other professions, recognizing that such sharing across disciplines can lead to a greater quality of biomedical research [21]. However, actual contribution of interdisciplinary approach cannot be evaluated based on this study.

Another limitation of the study could be attributed to differences in attrition rate between the cohorts. According to the School’s bylaws, the same regulations for student progress apply to undergraduate and PhD students. These regulations are in line with the Bologna accord but they were applied to PhD only in the TRIBE program, and this may have caused the differences in attrition rates between programs. However, the differences between the programs’ succession rates remained the same even when completion rate was calculated from the total number of enrolled students indicating that differences in attrition did not account for them.

Many factors have been suggested to account for the long duration of PhD studies. For example, PhD programs may be a source of revenue for institutions if students need to pay tuition to enroll into such a program; in that case schools are motivated to enroll more students and less motivated to pay attention to their successful completion. Additional contributing factors to inadequate completion rates and long duration of PhD studies may be insufficient supervision with no regulations and standards in place for proper supervision, lack of personal attachment to the course, separation of coursework and thesis research, no sense of belonging to a course, lack of quality and structure of PhD programs, or lack of a graduate school that provides supervision and structure to PhD programs [13]. Again, in the case of our USSM’s study, all conditions were similar for all programs except those innovations introduced in the TRIBE program.

The policies for the TRIBE program were based on the finding of low completion rates in the analysis conducted in 2011, interviews with students from the old PhD programs about their reasons for lack of success in the PhD program, analysis of curricula and policies of internationally renowned graduate programs and analysis of a theoretical framework related to quality assurance in education. According to the OECD report, the Croatian academic system suffers constraints in the development of quality assurance [22]. Although there has not been national implementation of a quality assurance framework, some institutions have introduced quality management standards [23]. The innovations in the TRIBE doctoral program were based on Deming’s theory of quality management [24, 25]. This supports the view that reforms to doctoral studies can only be achieved with clear and measurable criteria for students’ individual and overall program success. Without defining the success, it is impossible to evaluate the impact of any reforms aimed at improving PhD programs [26]. That is why we adopted Kirkpatrick’s four-level model [27] for educational research [28] and selected domains aimed at increasing completion rates and reducing time to degree, which can be considered as outcomes linked with hierarchically higher (or ‘better’, more desirable) outcomes (4th level).

The number and the impact factor of journals in which publications included in PhD theses were published did not appear to be influenced by our interventions. All three programs had similar numbers of publication per thesis and these were published in journals with similar impact factors. However, we are aware that journal impact factor is probably a poor surrogate for the quality of individual articles [29]. Indeed, the use of impact factors as a proxy for quality has been questioned many times. But it is unclear what should be used instead, and this measure is widely used to assess academic output [30]. For the purposes of this study we felt that citation analysis was inappropriate because it takes time for citations to accumulate, so reliable data will only be available for these cohorts in the future.

Our results suggest that students and their mentors are opting for the minimum requirements for a PhD thesis resulting in a small number of publications associated with each thesis, generally published in relatively low impact journals. We believe that significant improvement in quality and number of publications cannot be expected without raising the minimum requirement for publications constituting a PhD thesis.

One important problem that emerged during our analysis was the lack of availability of data related to the success of other comparable PhD programs in Croatia and Europe [31]. In the US, the Survey of Earned Doctorates (SED) has been conducted since 1957, providing an annual census of all individuals receiving research doctorates from accredited US institutions [32]. The SED collects information on the doctoral recipient’s educational history, demographic characteristics, and postgraduation plans. Results are used to assess characteristics of the doctoral population and trends in doctoral education and degrees.

Also, there are no publicly available data about time-to-degree in higher education in Croatia across different disciplines that we could use to describe the current status of undergraduate and graduate programs in Croatia. The only such study that we are aware of is our own work, published in 2012, in which we analyzed success and attrition rates of undergraduate students in the School of Medicine in Split, which revealed that the attrition was 26% over a 30-year studied period, with a trend towards improvement [33].

## Conclusions

In conclusion, our findings suggest that the implementation of progress reports, stricter regulations, selection criteria based on producing a feasible research plan, interdisciplinarity, education in complementary skills, and focus on developing a research plan was associated with higher graduation rates and significantly reduced time to degree in a medical PhD program. However, we need more studies about completion rates and time-to-degree in PhD programs of different institutions, as well as studies about other successful interventions in PhD education. Without transparency in doctoral education, comparisons between different institutions will remain impossible; therefore results cannot be analyzed and compared, improvements designed and applied, and thus educational programs may continue to have unacceptably poor performances.
